# Verification of the effects of calcium channel blockers on the immune microenvironment of breast cancer

**DOI:** 10.1186/s12885-019-5828-5

**Published:** 2019-06-24

**Authors:** Koji Takada, Shinichiro Kashiwagi, Yuka Asano, Wataru Goto, Katsuyuki Takahashi, Hisakazu Fujita, Tsutomu Takashima, Shuhei Tomita, Kosei Hirakawa, Masaichi Ohira

**Affiliations:** 1 Department of Surgical Oncology, Osaka City University Graduate School of Medicine, 1-4-3 Asahi-machi, Abeno-ku, Osaka, 545-8585 Japan; 2 Department of Pharmacology, Osaka City University Graduate School of Medicine, 1-4-3 Asahi-machi, Abeno-ku, Osaka, 545-8585 Japan; 3 Department of Scientific and Linguistic Fundamentals of Nursing, Osaka City University Graduate School of Nursing, 1-5-17 Asahi-machi, Abeno-ku, Osaka, 545-0051 Japan

**Keywords:** Calcium channel blockers, Breast cancer, Tumor-infiltrating lymphocytes, Immune microenvironment, Pre-operative chemotherapy

## Abstract

**Background:**

A higher density of tumor-infiltrating lymphocytes (TILs) can lead to greater therapeutic effects and improved prognoses in cancer treatment. Similar results have been observed in breast cancer, particularly in triple-negative breast cancer (TNBC) and human epidermal growth factor receptor 2-enriched breast cancer. Calcium channel blockers (CCBs) are antihypertensive drugs (AHTs) that have also been reported to suppress the functions of T cells and macrophages. In this study, we evaluated TILs before pre-operative chemotherapy (POC) in breast cancer and retrospectively analyzed the correlation between CCBs and TILs or prognosis.

**Methods:**

Of the patients treated with POC, 338 who had evaluable TILs were enrolled in this study. The correlations among TILs were evaluated according to standard methods, and CCB use and prognosis were investigated retrospectively.

**Results:**

Before POC, 65 patients (19.2%) took AHTs (CCBs: 41/338, 12.1%). The TIL density was significantly lower among patients administered CCBs for the group of all patients and for patients with TNBC (*p* = 0.040, *p* = 0.009, respectively). Additionally, patients with TNBC who were administered CCBs showed significantly lower response rates for POC (*p* = 0.040). In all patients receiving POC, no significant differences in disease-free survival (DFS) or overall survival (OS) were observed in patients administered CCBs (*p* = 0.712, *p* = 0.478, log-rank tests, respectively). Furthermore, no significant differences were found, even in patients with TNBC (DFS: *p* = 0.441, OS: *p* = 0.727, log-rank tests, respectively).

**Conclusions:**

In patients with TNBC undergoing treatment for hypertension with CCBs, TILs in the needle biopsy specimens before treatment were significantly lower, and the response rate of POC was not sufficient. Thus, the immunosuppressive effects of CCBs may also affect the immune microenvironment.

**Electronic supplementary material:**

The online version of this article (10.1186/s12885-019-5828-5) contains supplementary material, which is available to authorized users.

## Background

Although many drugs are used in the clinical setting, these drugs may cause unexpected side effects, including effects on immunity. For example, metformin, a drug prescribed for diabetes, activates CD8+ T cells [1]. Additionally, statins are hyperlipidemic drugs that have been reported to suppress immunity [2–5], and calcium channel blockers (CCBs) are antihypertensive drugs (AHTs) that have also been reported to suppress the functions of T cells and macrophages [6–12].

Immune responses around tumors are complex and can affect the therapeutic effects of chemotherapy and prognosis. Tumor-infiltrating lymphocytes (TILs), as indicators of the tumor microenvironment, affect the growth of cancer and the effects of chemotherapy. Therefore, a higher density of TILs can lead to greater therapeutic effects and improved prognoses, as has been observed in melanomas and lung cancer [13–15]. Similar results have been observed in breast cancer, particularly in triple-negative breast cancer (TNBC) and human epidermal growth factor receptor 2 (HER2)-enriched breast cancer (HER2BC) [16, 17]. Therefore, we hypothesized that CCBs may reduce the TIL density, thereby disrupting the therapeutic effects of drugs and worsening prognosis.

Accordingly, in this study, we evaluated TILs before pre-operative chemotherapy (POC) in patients with breast cancer and retrospectively analyzed the correlations between CCBs and TILs or prognosis.

## Methods

### Patient background

All patients who visited the Osaka City University Hospital from February 2007 to March 2018 were screened to obtain their medical histories. In cases of suspected breast cancer, core needle biopsy or vacuum-assisted biopsy was performed with ultrasonography (US). When diagnosed pathologically with breast cancer, the subtype of breast cancer was determined by immunostaining and staging with computed tomography (CT), US, and bone scintigraphy. If evaluation of metastasis to lymph nodes was difficult using these imaging tests, lymph node biopsy was performed. For immunostaining of samples, the expression levels of estrogen receptor (ER), progesterone receptor (PgR), HER2, and Ki67 were evaluated. The cut-off value for Ki-67 staining was set at 15% [18]. We defined ER−/PgR−/HER2+ breast cancer as HER2BC, ER−/PgR−/HER2- breast cancer as TNBC, and breast cancer that was not HER2BC or TNBC as luminal breast cancer (luminal BC) [19]. In total, 338 patients with breast cancer, diagnosed with stage IIA (T1, N1, M0 or T2, N0, M0), IIB (T2, N1, M0 or T3, N0, M0), IIIA (T1–2, N2, M0 or T3, N1–2, M0), IIIB (T4, N0–2, M0), or IIIC (T1–4, N3, M0), received POC. During the first half of the POC regimen, all patients received four courses of FEC100 (500 mg/m^2^ fluorouracil, 100 mg/m^2^ epirubicin, and 500 mg/m^2^ cyclophosphamide) every 3 weeks. During the second half of the POC regimen, 12 courses of 80 mg/m^2^ paclitaxel were administered to all patients weekly, and weekly (2 mg/kg) or tri-weekly (6 mg/kg) trastuzumab was also administered in cases of HER2-positive disease [20–22]. Antitumor effects were evaluated according to the Response Evaluation Criteria in Solid Tumors [23]. For analysis of the objective response rate (ORR), clinical partial response and complete response were defined as responders, and clinical stable disease and clinical progressive disease were defined as nonresponders. After confirming the therapeutic effects of POC, all patients were examined for continuation of AHTs before surgery; patients then underwent mastectomy or breast-conserving surgery [22]. Pathological complete response (pCR) was defined by the National Surgical Adjuvant Breast and Bowel Project B-18 protocol as “the complete disappearance of the invasive components of the lesion with or without intraductal components, including that in the lymph nodes” [24]. Standard postoperative radiotherapy was enforced if necessary, and postoperative adjuvant therapy suitable for the patient’s specific subtype was performed. As follow-up after surgery, all patients had physical examinations every 3 months, US every 6 months, and CT and bone scintigraphy annually. The median follow-up time was 1287 days (range, 13–3675 days) from operation.

### Histopathological evaluation of TIL density

Biopsy specimens before POC were used to evaluate TIL density. The definition and evaluation method of TILs were in accordance with the International TILs Working Group 2014 [25]. The average density of the infiltrating lymphocytes within the tumor stroma in five randomly selected fields was calculated. After categorization into four classes according to the TIL density (3: > 50%, 2: > 10–50%, 1: ≤ 10%, or 0: absent; Additional file 1: Figure S1), scores of 2 and 3 were defined as high, and scores of 0 and 1 were defined as low [26].

### Ethics statement

This study was conducted at Osaka City University Graduate School of Medicine, Osaka, Japan, according to the Reporting Recommendations for Tumor Marker Prognostic Studies (REMARK) guidelines and following a retrospectively written research, pathological evaluation, and statistical plan [27]. The study protocol was approved by the Ethics Committee of Osaka City University. Written informed consent was obtained from all patients (#926).

### Statistical methods

Correlations between the two groups were examined using chi-squared tests (or Fisher’s exact tests when necessary). Analysis of prognosis, such as disease free survival (DFS) or overall survival (OS), was carried out using the Kaplan-Meier method and log-rank tests. Hazard ratios (HRs) and 95% confidence intervals (CIs) were calculated using the Cox proportional hazards model, and multivariable analysis was analyzed in the Cox regression model. Statistical significance was assumed when the *p* values were less than 0.05. The JMP 11 software program (SAS, Tokyo, Japan) was used to analyze the data.

## Results

### Clinicopathological features and differences according to subtype

Three hundred thirty-eight patients received POC; the details of their clinicopathological features are summarized in Table 1. All patients were women, and the median age at operation was 55 years old (24–78 years old). The median tumor size was 28.7 mm (9.2–119.8 mm); the tumor size of 56 patients (16.6%) was 20 mm or less, and that of 44 patients (13.0%) was larger than 50 mm. Skin infiltration was observed in 50 patients (14.8%), and 224 patients (66.3%) were diagnosed with breast cancer having lymph node metastasis by imaging diagnosis. In classification by intrinsic subtype, 155 patients (45.9%) were classified as having luminal BC, 78 patients (23.1%) were classified as having HER2BC, and 105 patients (31.1%) were classified as having TNBC. Moreover, 298 patients (88.2%) were evaluated as responders in ORR. In the pathological examination of surgical specimens, 116 patients (34.3%) showed pCR. By evaluating the biopsy specimens before POC, 158 patients (46.7%) were classified into the high TIL density group, and 180 patients (53.3%) were classified in the low TIL density group.

**Table 1:** Clinicopathological features of 338 patients who were treated with preoperative chemotherapy

Parameters (*n* = 338)	Number of patients (%)
Age (years old)	55 (24–78)
Tumor size (mm)	28.7 (9.2–119.8)
Skin infiltration	
Negative / Positive	288 (85.2%) / 50 (14.8%)
Lymph node metastasis	
N0 / N1 / N2 / N3	114 (33.7%) / 128 (37.9%) / 63 (18.6%) / 33(9.8%)
Estrogen receptor	
Negative / Positive	187 (55.3%) / 151 (44.7%)
Progesterone receptor	
Negative / Positive	236 (69.8%) / 102 (30.2%)
HER2	
Negative / Positive	214 (63.3%) / 124 (36.7%)
Ki67	
≤ 15% / > 15%	105 (31.1%) / 233 (68.9%)
Intrinsic subtype	
Luminal BC / HER2BC / TNBC	155 (45.8%) / 78 (23.1%) / 105 (31.1%)
Objective response rate	
Non-Responders / Responders	40 (11.8%) / 298 (88.2%)
Pathological response	
Non-pCR / pCR	222 (65.7%) / 116 (34.3%)
TILs	
Low / High	180 (53.3%) / 158 (46.7%)
Hypertension	
No / Yes	273 (80.8%) / 65 (19.2%)
Number of medicine types for hypertension	
0 / 1 / 2 / 3	273 (80.8%) / 41 (12.1%) / 21 (6.2%) / 2 (0.6%) / 1 (0.3%)
Calcium channel blockers	
No / Yes	297 (87.9%) / 41 (12.1%)
ACEi or ARBs	
No / Yes	305 (90.2%) / 33 (9.8%)
Beta-blockers	
No / Yes	326 (96.4%) / 12 (3.6%)
Diuretics	
No / Yes	331 (97.9%) / 7 (2.1%)

*HER* human epidermal growth factor receptor, *Luminal BC* luminal breast cancer, *HER2BC* human epidermal growth factor receptor 2-enriched breast cancer, *TNBC* triple-negative breast cancer, *pCR* pathological complete response, *TILs* tumor- infiltrating lymphocytes, *AHT* antihypertensive drug, *ACEi* angiotensin-converting-enzyme inhibitors, *ARBs* angiotensin II receptor blockers

Before POC, 65 patients (19.2%) took AHTs. Patients who had been treated before the first visit but were not treated before POC were divided into groups excluding hypertensive patients. There were no untreated patients with hypertension before POC. The following AHTs were administered: CCBs, angiotensin-converting-enzyme inhibitor, angiotensin II receptor blockers, beta-blockers, and diuretics. Forty-one patients (12.1%) were taking CCBs, and CCBs were the most commonly used medication for hypertension. Twenty-four patients (7.1%) took several medications for hypertension. No patients started new AHTs or needed additional AHTs during POC. In three patients (0.9%), AHTs were discontinued during POC. Both of these patients were taking CCBs only, and the times of discontinuation were 1.5 months, 1 month, and 10 days before surgery, respectively.

Comparison of clinicopathological features based on intrinsic subtypes showed poorer pathological response in luminal BC than in HER2BC or TNBC (luminal BC: 18.1%, HER2BC: 55.1%, TNBC: 42.9%; Additional file 2: Table S1). For age, we set the median as the cutoff value. In luminal BC, the rate of patients in the high TIL density group was lower than those in patients with other subtypes (luminal BC: 30.3%, HER2BC: 67.9%, TNBC: 55.2%). There were no significant differences in other items by subtype.

### Differences in clinicopathological features due to TILs or hypertension treatment

We examined differences in clinicopathological features due to TILs (Additional file 3: Table S2). In the high TIL density group (*n* = 338), the expression levels of ER and PgR were significantly lower (*p* < 0.001 and *p* < 0.001, respectively), whereas the expression levels of HER2 and Ki67 were significantly higher than in the low TIL density group (*p* = 0.023, *p* < 0.001, respectively). Moreover, the TIL density was significantly lower in luminal BC and significantly higher in HER2BC and TNBC (*p* < 0.001, *p* < 0.001, *p* < 0.001, respectively). The ORR and pCR were significantly higher in the high TIL density group than in the low TIL density group (*p* < 0.001, *p* < 0.001, respectively). In 105 patients with TNBC and 78 patients with HER2BC, the same correlation between TILs and ORR or pCR was found (TNBC: *p* = 0.008, *p* = 0.042; HER2BC: *p* = 0.017, *p* = 0.019, respectively).

Notably, patients administered CCBs had significantly lower TIL densities (*p* = 0.040). Furthermore, in patients with TNBC, the TIL density was significantly lower in patients receiving hypertension treatment and patients receiving CCBs (*p* = 0.003, *p* = 0.009, respectively). In HER2BC, there were no correlations between AHTs and TILs.

The correlations between CCBs and clinicopathological features were examined in chi-squared tests and are shown in Table 2. In all patients and in patients with TNBC, patients administered CCBs were significantly older than patients without CCB administration (*p* < 0.001, *p* = 0.004, respectively). Moreover, patients with TNBC who were administered CCBs showed significantly lower response rates for POC (*p* = 0.040). No correlations between CCBs and pCRs was observed (*p* = 0.649). However, when we focused on patients with hypertension only, no relationship was found between CCBs and TILs (Additional file 4: Table S3).

**Table 2:** Difference in clinicopathological features due to calcium channel blockers^a^

Parameters	All case (*n* = 338)			TNBC (*n* = 105)			HER2BC (*n* = 78)		
	Calcium channel blockers		*p* value	Calcium channel blockers		*p* value	Calcium channel blockers		*p* value
	No (*n* = 297)	Yes (*n* = 41)		No (*n* = 94)	Yes (*n* = 11)		No (*n* = 68)	Yes (*n* = 10)	
Age (years old)									
≤ 55	170 (57.2%)	7 (17.1%)	< 0.001	59 (62.8%)	2 (18.2%)	0.004	27 (39.7%)	2 (20.0%)	0.234
> 55	127 (42.8%)	34 (82.9%)		35 (37.2%)	9 (81.8%)		41 (60.3%)	8 (80.0%)	
Tumor size (mm)									
≤ 50	258 (86.9%)	36 (87.8%)	0.868	82 (87.2%)	8 (72.7%)	0.197	60 (88.2%)	10 (100.0%)	0.258
> 50	39 (13.1%)	5 (12.2%)		12 (12.8%)	3 (27.3%)		8 (11.8%)	0 (0.0%)	
Skin infiltration									
Negative	255 (85.9%)	33 (80.5%)	0.365	86 (91.5%)	8 (72.7%)	0.055	58 (85.3%)	9 (90.0%)	0.694
Positive	42 (14.1%)	8 (19.5%)		8 (8.5%)	3 (27.3%)		10 (14.7%)	1 (10.0%)	
Lymph node status									
Negative	102 (34.3%)	12 (29.3%)	0.887	28 (29.8%)	4 (36.4%)	0.658	28 (41.2%)	4 (40.0%)	0.945
Positive	195 (65.7%)	29 (70.7%)		66 (70.2%)	7 (63.6%)		40 (58.8%)	6 (60.0%)	
Estrogen receptor									
Negative	166 (55.9%)	21 (51.2%)		–	–	–	–	–	–
Positive	131 (44.1%)	20 (48.8%)	0.574	–	–		–	–	
Progesterone receptor									
Negative	207 (69.7%)	29 (70.7%)	0.893	–	–	–	–	–	–
Positive	90 (30.3%)	12 (29.3%)		–	–		–	–	
HER2									
Negative	190 (64.0%)	24 (58.5%)	0.500	–	–		–	–	–
Positive	107 (36.0%)	17 (41.5%)		–	–		–	–	
Ki67									
≤ 15%	87 (29.3%)	18 (43.9%)	0.058	16 (17.0%)	2 (18.2%)	0.924	15 (22.1%)	5 (50.0%)	0.060
> 15%	210 (70.7%)	23 (56.1%)		78 (83.0%)	9 (81.8%)		53 (77.9%)	5 (50.0%)	
Intrinsic subtype Luminal BC									
HER2BC, TNBC	162 (54.5%)	21 51.2%)	0.690	–	–		–	–	–
Luminal BC	135 (45.5%)	20 (48.8%)		–	–		–	–	
Intrinsic subtype HER2BC									
Luminal BC, TNBC	229 (77.1%)	31 (75.6%)	0.832	–	–		–	–	–
HER2BC	68 (22.9%)	10 (24.4%)		–	–		–	–	
Intrinsic subtype TNBC									
Luminal BC, HER2BC	203 (68.4%)	30 (73.2%)	0.5332	–	–		–	–	–
TNBC	94 (31.6%)	11 (26.8%)		–	–		–	–	
Objective response rate									
Non-Responders	32 (10.8%)	8 (19.5%)	0.105	12 (12.8%)	4 (36.4%)	0.040	5 (7.4%)	0 (0.0%)	0.382
Responders	265 (89.2%)	33 (80.5%)		82 (87.2%)	7 (63.6%)		63 (92.6%)	10 (100.0%)	
Pathological response									
Non-pCR	195 (65.7%)	27 (65.9%)	0.980	53 (56.4%)	7 (63.6%)	0.649	33 (48.5%)	2 (20.0%)	0.093
pCR	102 (34.3%)	14 (34.1%)		41 (43.6%)	4 (36.4%)		35 (51.5%)	8 (80.0%)	
TILs									
Low	152 (51.2%)	28 (68.3%)	0.040	38 (40.4%)	9 (81.8%)	0.009	22 (32.4%)	3 (30.0%)	0.884
High	145 (48.8%)	13 (31.7%)		56 (59.6%)	2 (18.2%)		46 (67.6%)	7 (70.0%)	
Hypertension									
No	273 (91.9%)	0 (0.0%)	< 0.001	90 (95.7%)	0 (0.0%)	< 0.001	60 (88.2%)	0 (0.0%)	< 0.001
Yes	24 (8.1%)	41 (100.0%)		4 (4.3%)	11 (100.0%)		8 (11.8%)	10 (100.0%)	
Multiple types of AHT									
No	293 (98.6%)	21 (51.2%)	< 0.001	93 (98.9%)	6 (54.5%)	< 0.001	67 (98.5%)	4 (40.0%)	< 0.001
Yes	4 (1.4%)	20 (48.8%)		1 (1.1%)	5 (45.5%)		1 (1.5%)	6 (60.0%)	
ACEi or ARBs									
No	281 (94.6%)	24 (58.5%)	< 0.001	91 (96.8%)	8 (72.7%)	0.001	62 (91.2%)	4 (40.0%)	< 0.001
Yes	16 (5.4%)	17 (41.5%)		3 (3.2%)	3 (27.3%)		6 (8.8%)	6 (60.0%)	
Beta-blockers									
No	289 (97.3%)	37 (90.2%)	0.022	93 (98.9%)	3 (27.3%)	< 0.001	66 (97.1%)	10 (100.0%)	0.589
Yes	8 (2.7%)	4 (9.8%)		1 (1.1%)	8 (72.7%)		2 (2.9%)	0 (0.0%)	
Diuretics									
No	293 (98.6%)	38 (92.7%)	0.012	93 (98.9%)	10 (90.9%)	0.066	67 (98.5%)	10 (100.0%)	0.704
Yes	4 (1.4%)	3 (7.3%)		1 (1.1%)	1 (9.1%)		1 (1.5%)	0 (0.0%)	

*HER* human epidermal growth factor receptor, *Luminal BC* luminal breast cancer, *HER2BC* human epidermal growth factor receptor 2-enriched breast cancer, *TNBC* triple-negative breast cancer, *pCR* pathological complete response, *TILs* tumor- infiltrating lymphocytes, *AHT* antihypertensive drug, *ACEi* angiotensin-converting-enzyme inhibitors, *ARBs* angiotensin II receptor blockers

^a^Correlations between the two groups were examined in chi-squared tests

### Influence of CCBs on DFS and OS

**Fig. 1:**
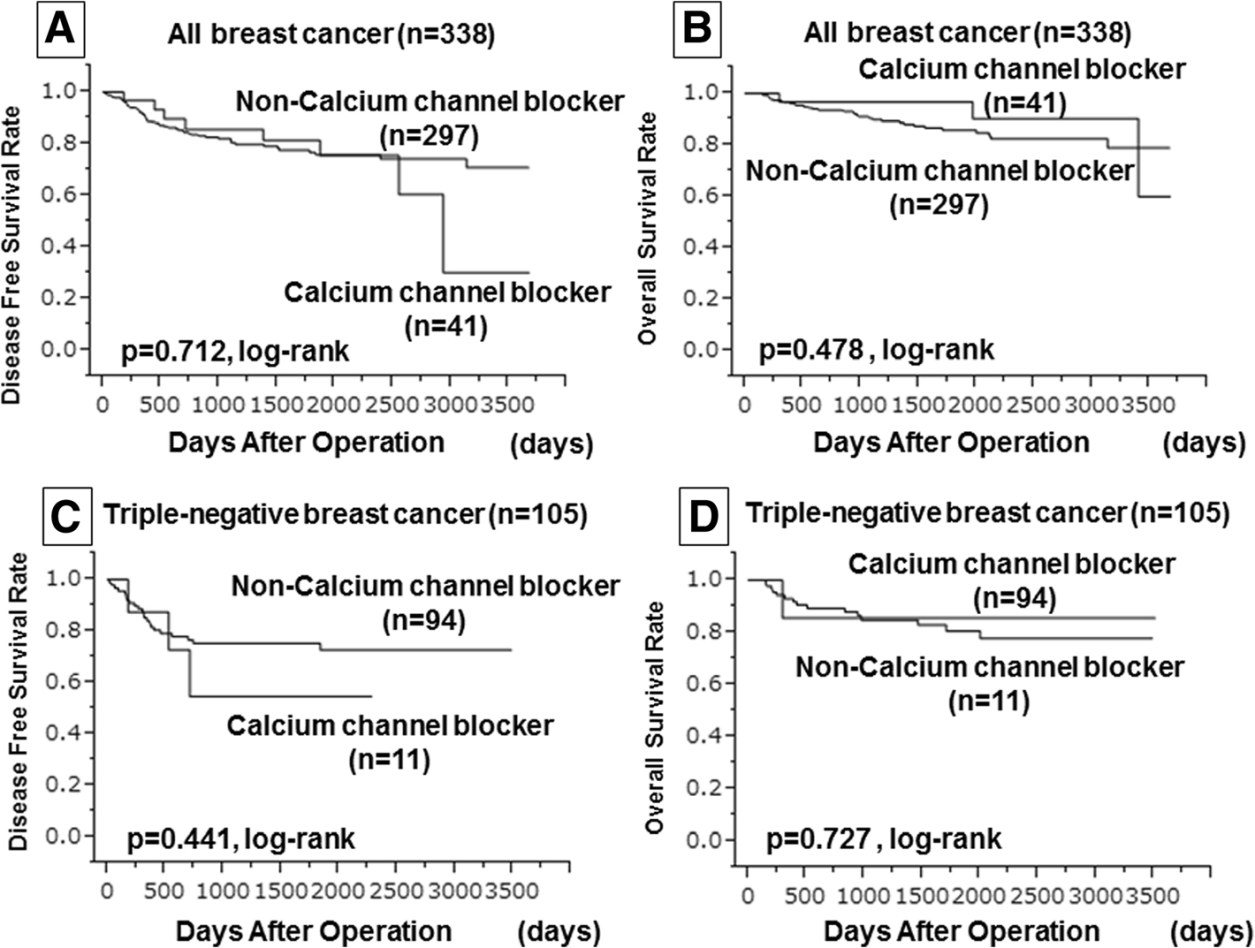
Comparison of disease-free survival (DFS) and overall survival (OS) using the Kaplan-Meier method based on the presence or absence of calcium channel blockers (CCBs). DFS (**a**) and OS (**b**). DFS (**c**) and OS (**d**) in patients with triple-negative breast cancer (TNBC)

In all patients receiving POC, no significant differences in DFS or OS were observed due to the use of CCBs, as determined using the Kaplan-Meier method and log-rank tests (*p* = 0.712, *p* = 0.478, log-rank tests, respectively; Fig. 1a, b). Furthermore, no significant differences were found, even in patients with TNBC (DFS: *p* = 0.441, OS: *p* = 0.727, log-ranks, respectively; Fig. 1c, d).

In patients with TNBC, a high TIL density significantly contributed to longer DFS using univariate analysis (*p* = 0.004, HR = 0.306; Table 3). Additionally, in multivariate analysis with DFS and OS, response in ORR was an independent factor (DFS: *p* = 0.004, HR = 0.258; OS: *p* = 0.001, HR = 0.143; Tables 3 and 4). Despite these results, there were no significant differences in univariate analysis with DFS or OS due to CCBs (DFS: *p* = 0.472, HR = 1.601; OS: *p* = 0.715, HR = 0.699). Similar analyses were carried out for all breast cancer and HER2BC, but no significant differences were found (Additional file 5, 6, 7, 8: Table S4–7).

**Table 3:** Univariate and multivariate analysis with respect to DFS in TNBC

Parameters	Univarite analysis			Multivariate analysis		
	Hazard ratio	95% CI	*p* value	Hazard ratio	95% CI	*p* value
Age at opetation (yr)						
≤ 55 vs > 55	0.758	0.320–1.682	0.501			
Tumor size (mm)						
≤ 50 vs > 50	2.718	1.056–6.245	0.039	1.264	0.464–3.165	0.630
Skin infiltration						
Negative vs Positive	2.349	0.781–5.806	0.118			
Lymph node status						
Negative vs Positive	2.664	0.922–11.261	0.073	1.954	0.634–8.522	0.263
Ki67						
≤ 15% vs > 15%	1.700	0.587–7.197	0.359			
Objective response rate						
Non-Responders vs Responders	0.146	0.065–0.342	< 0.001	0.258	0.106–0.638	0.004
Pathological response						
Non-pCR vs pCR	0.207	0.060–0.545	0.001	0.395	0.108–1.166	0.095
TILs						
Low vs High	0.306	0.125–0.689	0.004	0.464	0.180–1.120	0.088
Hypertension						
No vs Yes	2.212	0.735–5.476	0.145			
Multiple types of AHT						
No vs Yes	1.309	0.210–4.431	0.725			
Calcium channel blockers						
No vs Yes	1.601	0.379–4.627	0.472			
ACEi or ARBs						
No vs Yes	1.554	0.249–5.265	0.574			
Beta-blockers						
No vs Yes	0.894	0.050–4.229	0.911			
Diuretics						
No vs Yes	2.850	0.159–13.551	0.378			

*DFS* Disease-free survival, *TNBC* triple-negative breast cancer, *CI* confidence intervals, *pCR* pathological complete response, *TILs* tumor- infiltrating lymphocytes, *AHT* antihypertensive drug, *ACEi* angiotensin-converting-enzyme inhibitors, *ARBs* angiotensin II receptor blockers

**Table 4:** Univariate and multivariate analysis with respect to OS in TNBC

Parameters	Univarite analysis			Multivariate analysis		
	Hazard ratio	95% CI	*p* value	Hazard ratio	95% CI	*p* value
Age at opetation (yr)						
≤ 55 vs > 55	0.581	0.182–1.603	0.302			
Tumor size (mm)						
≤ 50 vs > 50	2.366	0.661–6.800	0.168			
Skin infiltration						
Negative vs Positive	2.948	0.822–8.488	0.091	3.321	0.891–10.307	0.071
Lymph node status						
Negative vs Positive	2.269	0.631–14.474	0.233			
Ki67						
≤ 15% vs > 15%	3.762	0.756–68.181	0.120			
Objective response rate						
Non-Responders vs Responders	0.090	0.032–0.244	< 0.001	0.143	0.045–0.430	0.001
Pathological response						
Non-pCR vs pCR	0.074	0.004–0.365	< 0.001	0.143	0.008–0.799	0.024
TILs						
Low vs High	0.411	0.140–1.109	0.079	0.903	0.288–2.654	0.855
Hypertension						
No vs Yes	1.161	0.182–4.181	0.847			
Multiple types of AHT						
No vs Yes	0.928	0.051–4.609	0.942			
Calcium channel blockers						
No vs Yes	0.699	0.039–3.465	0.715			
ACEi or ARBs						
No vs Yes	1.161	0.064–5.759	0.887			
Beta-blockers						
No vs Yes	–	–	0.206			
Diuretics						
No vs Yes	4.138	0.228–20.631	0.258			

*OS* Overall survival, *TNBC* triple-negative breast cancer, *CI* confidence intervals, *pCR* pathological complete response, *TILs* tumor- infiltrating lymphocytes, *AHT* antihypertensive drug, *ACEi* angiotensin-converting-enzyme inhibitors, *ARBs* angiotensin II receptor blockers

## Discussion

In previous studies, CCBs have been shown to inhibit apoptosis by interfering with calcium-triggered signals, suggesting the possibility of promoting cancer [28]. Accordingly, numerous studies have been conducted on the risk of developing breast cancer by CCBs [29, 30]. A recent meta-analysis of observational studies has reported that there is no correlation between CCBs and carcinogenesis in breast cancer (risk ratio: 1.07, 95% CI: 0.99–1.16) [29]. In contrast, some reports have shown that CCBs suppress the activity of T cells by inhibiting interleukin-2, which is required for the differentiation of T cells [6, 8, 9, 31].

In this study, we evaluated the correlations of TILs with hypertension and AHTs and showed, for the first time, that the TIL density was decreased by CCBs. This result suggested that CCBs may also affect the immune TME (iTME). In particular, in patients with TNBC, responders in ORR decreased as the TIL density decreased, consistent with our hypothesis. Nonetheless, CCBs did not affect prognosis. We speculated that this result could be related to changes in the ratios of TIL subsets. TILs contain various subsets, some of which suppress the growth of cancer, and some of which promote cancer progression [25]. In one study, the concentration of CCBs that suppressed T cells differed depending on the T-cell type; CD4-positive T cells were suppressed at lower CCB concentrations than CD8-positive T cells [7]. Additionally, many reports have shown that increased numbers of CD8-positive T cells in the iTME are an indicator of improved prognosis [32, 33]. In contrast, other reports have shown that increased numbers of CD4-positive T cells in the iTME can be related to either an improved or worsened prognosis [32, 34, 35]. The poor prognosis could be explained by the observation that CD4 is expressed in most regulatory T cells that promote cancer progression. We have previously reported that the CD8 to FOXP3 lymphocyte ratio in the iTME affects the therapeutic outcomes and prognosis of patients with TNBC and HER2BC who received POC [36]. However, the strength of the inhibitory effect on T cells varies depending on the type of CCB [9]. Furthermore, macrophages also play a major role in the iTME and are suppressed by CCBs [10, 31, 37]. In this study, we did not analyze the type and dose of CCBs; thus, these drugs may have affected the ratio of TIL subsets and thereby influenced prognosis.

This study was limited by the fa13ct that we did not evaluate the different types and doses of AHTs used. Moreover, it was not known when or for how long patients were taking AHTs before POC. In other words, changes over time due to CCBs were unclear. After operation, it is unknown how treatment for hypertension was performed. However, our data strongly supported that CCBs influenced the iTME. Depending on the method for using CCBs, iTME may be exacerbated, which may lead to a poor prognosis. In contrast, if our hypothesis is correct and we can further suppress TILs that promote cancer by adjusting CCBs, we may be able to improve prognoses. Indeed, we previously reported that the iTME affects prognosis after recurrence [38]. Therefore, in future studies, we plan to evaluate changes in the iTME during treatment and assess the influence of CCBs on iTME.

## Conclusions

In patients with TNBC undergoing treatment with CCBs for hypertension, TILs in the needle biopsy specimens before treatment were significantly lower, and the response rate of POC was not effective. These results suggested that immunosuppressive action by CCBs may affect not only lymphocytes in the blood but also lymphocytes in the immune microenvironment.

## Additional files

Additional file 1: **Figure S1.** Histopathological evaluation of TILs. TIL density was evaluated in biopsy specimens by core needle biopsy or vacuum-assisted biopsy taken before pre-operative chemotherapy. Five random fields were evaluated. (A) > 50%: score 3, (B) > 10–50%: score 2, (C) ≤ 10%: score 1, (D) absent: score 0. (PPTX 2000 kb)

Additional file 2: **Table S1.** Clinicopathological features by subtype. (DOCX 21 kb)

Additional file 3: **Table S2.** Difference in clinicopathological features due to TILs. (DOCX 24 kb)

Additional file 4: **Table S3.** Difference in clinicopathological features due to calcium channel blockers in hypertension patients. (DOCX 25 kb)

Additional file 5: **Table S4.** Univariate and multivariate analysis with respect to DFS. (DOCX 22 kb)

Additional file 6: **Table S5.** Univariate and multivariate analysis with respect to DFS in HER2BC. (DOCX 21 kb)

Additional file 7: **Table S6.** Univariate and multivariate analysis with respect to OS. (DOCX 22 kb)

Additional file 8: **Table S7.** Univariate and multivariate analysis with respect to OS in HER2BC. (DOCX 21 kb)

## Data Availability

The data and materials used and analyzed in the current study would be available from the corresponding author on request.

## Abbreviations

AHTs: Antihypertensive drugs
CCBs: Calcium channel blockers
CIs: Confidence intervals
CT: Computed tomography
DFS: Disease-free survival
ER: Estrogen receptor
HER2: Human epidermal growth factor receptor 2
HER2BC: Human epidermal growth factor receptor 2-enriched breast cancer
HR: Hazard ratio
iTME: Immune tumor microenvironment
Luminal BC: Hormone receptor-positive breast cancer
ORR: Objective response rate
OS: Overall survival
pCR: Pathological complete response
PgR: Progesterone receptor
POC: Pre-operative chemotherapy
REMARK: Reporting Recommendations for Tumor Marker Prognostic Studies
TILs: Tumor-infiltrating lymphocytes
TNBC: Triple-negative breast cancer
US: Ultrasonography
