# Supplementary figures and images for: Verification of the effects of calcium channel blockers on the immune microenvironment of breast cancer
Source: BMC Cancer. 2019 Jun 24;19:615. doi: 10.1186/s12885-019-5828-5 (PMC6591916; doi:10.1186/s12885-019-5828-5)

## Slide 1
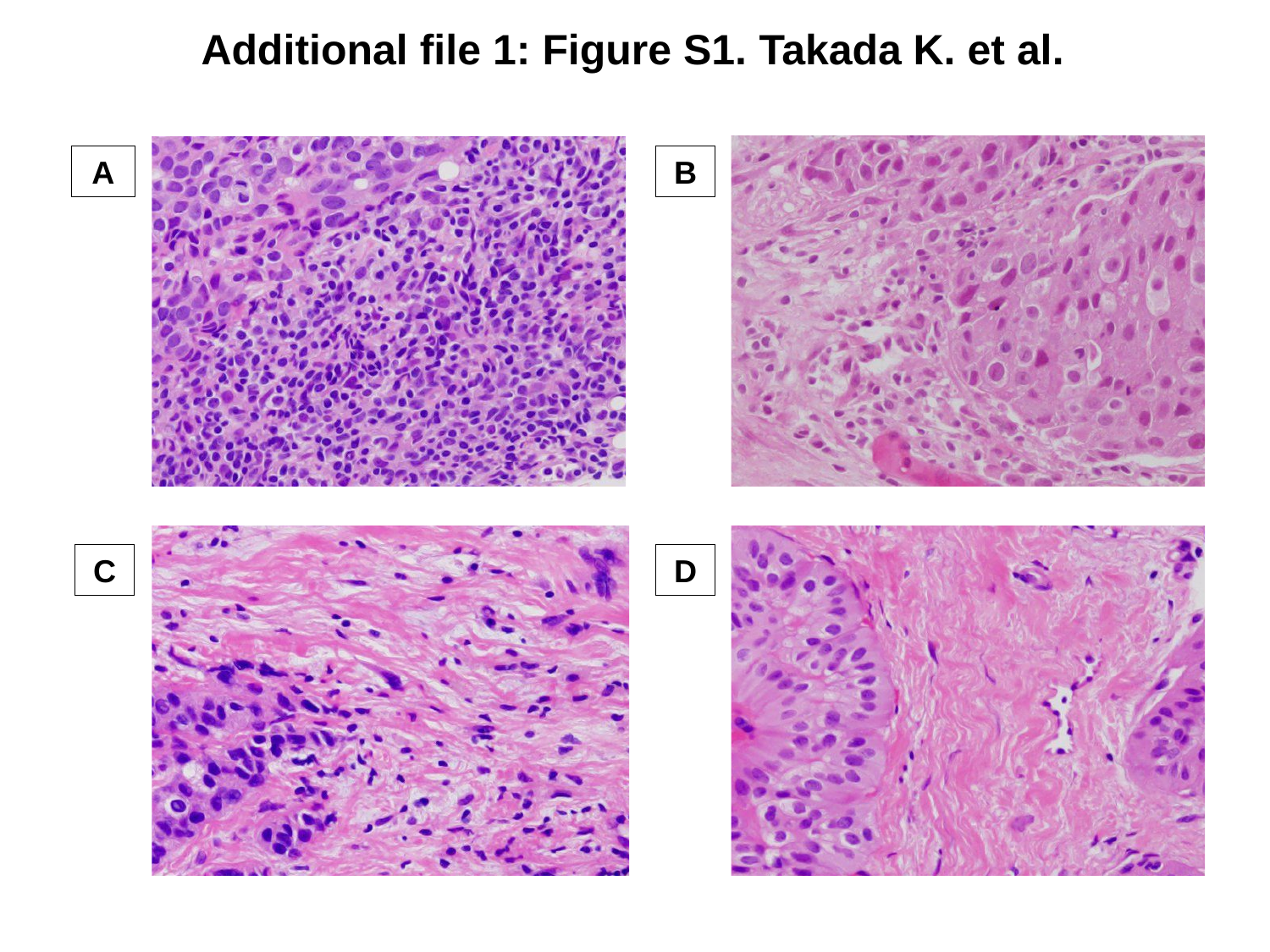

Additional file 1: Figure S1. Takada K. et al.
A
B
C
D

Supplement: Supplementary file 1 — Figure S1. Histopathological evaluation of TILs. TIL density was evaluated in biopsy specimens by core needle biopsy or vacuum-assisted biopsy taken before pre-operative chemotherapy. Five random fields were evaluated. (A) > 50%: score 3, (B) > 10–50%: score 2, (C) ≤ 10%: score 1, (D) absent: score 0. (PPTX 2000 kb) [file 12885_2019_5828_MOESM1_ESM.pptx]
