# Supplementary material for: Verification of the effects of calcium channel blockers on the immune microenvironment of breast cancer
Source: BMC Cancer. 2019 Jun 24;19:615. doi: 10.1186/s12885-019-5828-5 (PMC6591916; doi:10.1186/s12885-019-5828-5)
Supplement: Supplementary file 2 — Table S1. Clinicopathological features by subtype. (DOCX 21 kb) [file 12885_2019_5828_MOESM2_ESM.docx]

**Additional file 2: Table S1.** **Clinicopathological features by subtype**

| Parameters | Intrinsic subtype | | |
| --- | --- | --- | --- |
|  | Luminal BC  (*n* = 155) | HER2BC  (*n* = 78) | TNBC  (*n* =105) |
| Age (years old)  ≤ 55  > 55 | 87 (56.1%)  68 (43.9%) | 29 (37.2%)  49 (62.8%) | 61 (58.1%)  44 (41.9%) |
| Tumor size (mm)  ≤ 50  > 50 | 134 (86.5%)  21 (13.5%) | 70 (89.7%)  8 (10.3%) | 90 (85.7%)  15 (14.3%) |
| Skin infiltration  Negative  Positive | 127 (81.9%)  28 (18.1%) | 67 (85.9%)  11 (14.1%) | 94 (89.5%)  11 (10.5%) |
| Lymph node status  Negative  Positive | 50 (32.3%)  105 (67.7%) | 32 (41.0%)  46 (59.0%) | 32 (30.5%)  73 (69.5%) |
| Estrogen receptor  Negative  Positive | 4 (2.6%)  151 (97.4%) | 78 (100.0%)  0 (0.0%) | 105 (100.0%)  0 (0.0%) |
| Progesterone receptor  Negative  Positive | 53 (34.2%)  102 (65.8%) | 78 (100.0%)  0 (0.0%) | 105 (100.0%)  0 (0.0%) |
| HER2  Negative  Positive | 109 (29.7%)  46 (70.3%) | 0 (0.0%)  78 (100.0%) | 105 (100.0%)  0 (0.0%) |
| Ki67  ≤15 %  >15 % | 67 (43.2%)  88 (56.8%) | 20 (25.6%)  58 (74.4%) | 18 (17.1%)  87 (82.9%) |
| Objective response rate  Non-Responders  Responders | 19 (12.3%)  136 (87.7%) | 5 (6.4%)  73 (93.6%) | 16 (15.2%)  89 (84.8%) |
| Pathological response  Non-pCR  pCR | 127 (81.9%)  28 (18.1%) | 35 (44.9%)  43 (55.1%) | 60 (57.1%)  45 (42.9%) |
| TILs  Low  High | 108 (69.7%)  47 (30.3%) | 25 (32.1%)  53 (67.9%) | 47 (44.8%)  58 (55.2%) |
| Hypertension  No  Yes | 123 (79.4%)  32 (20.6%) | 60 (76.9%)  18 (23.1%) | 90 (85.7%)  15 (14.3%) |
| Multiple types of AHT  No  Yes | 144 (92.9%)  11 (7.1%) | 71 (91.0%)  7 (9.0%) | 99 (94.3%)  6 (5.7%) |
| Calcium channel blockers  No  Yes | 135 (87.1%)  20 (12.9%) | 68 (87.2%)  10 (12.8%) | 94 (89.5%)  11 (10.5%) |
| ACEi or ARBs  No  Yes | 140 (90.3%)  15 (9.7%) | 66 (84.6%)  12 (15.4%) | 99 (94.3%)  6 (5.7%) |
| Beta-blockers  No  Yes | 149 (96.1%)  6 (3.9%) | 76 (97.4%)  2 (2.6%) | 101 (96.2%)  4 (3.8%) |
| Diuretics  No  Yes | 151 (97.4%)  4 (2.6%) | 77 (98.7%)  1 (1.3%) | 103 (98.1%)  2 (1.9%) |

HER: human epidermal growth factor receptor. Luminal BC, luminal breast cancer HER2BC, human epidermal growth factor receptor 2-enriched breast cancer. TNBC, triple-negative breast cancer. pCR, pathological complete response. TILs: tumor- infiltrating lymphocytes. AHT: antihypertensive drug. ACEi: angiotensin-converting-enzyme inhibitors, ARBs: angiotensin II receptor blockers.
