# Supplementary material for: Verification of the effects of calcium channel blockers on the immune microenvironment of breast cancer
Source: BMC Cancer. 2019 Jun 24;19:615. doi: 10.1186/s12885-019-5828-5 (PMC6591916; doi:10.1186/s12885-019-5828-5)
Supplement: Supplementary file 3 — Table S2. Difference in clinicopathological features due to TILs. (DOCX 24 kb) [file 12885_2019_5828_MOESM3_ESM.docx]

**Additional file 3: Table S2.** **Difference in clinicopathological features due to TILs***

| Parameters | All case (*n* = 338) | | | TNBC (*n* =105) | | | HER2BC (*n* =78) | | |
| --- | --- | --- | --- | --- | --- | --- | --- | --- | --- |
|  | TILs | | *p* value | TILs | | *p* value | TILs | | *p* value |
|  | Low (*n* =180) | High (*n* =158) |  | Low (*n* =47) | High (*n* =58) |  | Low (*n* =25) | High (*n* =53) |  |
| Age (years old)  ≤ 55  > 55 | 92 (51.1%)  88 (48.9%) | 85 (53.8%)  73 (46.2%) | 0.623 | 24 (51.1%)  23 (48.9%) | 37 (63.8%)  21 (36.2%) | 0.192 | 9 (36.0%)  16 (64.0%) | 20 (37.7%)  33 (62.3%) | 0.884 |
| Tumor size (mm)  ≤ 50  > 50 | 153 (85.0%)  27 (15.0%) | 141 (89.2%)  17 (10.8%) | 0.249 | 37 (78.7%)  10 (21.3%) | 53 (91.4%)  5 (8.6%) | 0.066 | 21 (84.0%)  4 (16.0%) | 49 (92.5%)  4 (7.5%) | 0.257 |
| Skin infiltration  Negative  Positive | 145 (80.6%)  35 (19.4%) | 143 (90.5%)  15 (9.5%) | 0.010 | 40 (85.1%)  7 (14.9%) | 54 (93.1%)  4 (6.9%) | 0.189 | 18 (72.0%)  7 (28.0%) | 49 (92.5%)  4 (7.5%) | 0.015 |
| Lymph node status  Negative  Positive | 58 (32.2%)  122 (67.8%) | 56 (35.4%)  102 (64.6%) | 0.648 | 14 (29.8%)  33 (70.2%) | 18 (31.0%)  40 (69.0%) | 0.892 | 7 (28.0%)  18 (72.0%) | 25 (47.2%)  28 (52.8%) | 0.110 |
| Estrogen receptor  Negative  Positive | 74 (41.1%)  106 (58.9%) | 113 (71.5%)  45 (28.5%) | <0.001 | -  - | -  - | - | -  - | -  - | - |
| Progesterone receptor  Negative  Positive | 106 (58.9%)  74 (41.1%) | 130 (82.3%)  28 (17.7%) | <0.001 | -  - | -  - | - | -  - | -  - | - |
| HER2  Negative  Positive | 124 (68.9%)  56 (31.1%) | 90 (57.0%)  68 (43.0%) | 0.023 | -  - | -  - | - | -  - | -  - | - |
| Ki67  ≤15 %  >15 % | 73 (40.6%)  107 (59.4%) | 32 (20.3%)  126 (79.7%) | <0.001 | 16 (34.0%)  31 (66.0%) | 2 (3.5%)  56 (96.5%) | <0.001 | 7 (28.0%)  18 (72.0%) | 40 (75.5%)  13 (24.5%) | 0.747 |
| Intrinsic subtype Luminal BC  HER2BC, TNBC  Luminal BC | 72 (40.0%)  108 (60.0%) | 111 (70.3%)  47 (29.7%) | <0.001 | -  - | -  - | - | -  - | -  - | - |
| Intrinsic subtype HER2BC  Luminal BC, TNBC  HER2BC | 155 (86.1%)  25 (13.9%) | 105 (66.5%)  53 (33.5%) | <0.001 | -  - | -  - | - | -  - | -  - | - |
| Intrinsic subtype TNBC  Luminal BC, HER2BC  TNBC | 133 (73.9%)  47 (26.1%) | 100 (63.3%)  58 (36.7%) | 0.036 | -  - | -  - | - | -  - | -  - | - |
| Objective response rate  Non-Responders  Responders | 32 (17.8%)  148 (82.2%) | 8 (5.1%)  150 (94.9%) | <0.001 | 13 (27.7%)  34 (72.3%) | 3 (5.2%)  55 (94.8%) | 0.008 | 4 (16.0%)  21 (84.0%) | 1 (1.9%)  52 (98.1%) | 0.017 |
| Pathological response  Non-pCR  pCR | 141 (78.3%)  39 (21.7%) | 81 (51.3%)  77 (48.7%) | <0.001 | 32 (68.1%)  15 (31.9%) | 28 (48.3%)  30 (51.7%) | 0.042 | 9 (36.0%)  16 (64.0%) | 19 (35.8%)  34 (64.2%) | 0.019 |
| Hypertension  No  Yes | 139 (77.2%)  41 (22.8%) | 134 (84.8%)  24 (15.2%) | 0.078 | 35 (74.5%)  12 (25.5%) | 55 (94.8%)  3 (5.2%) | 0.003 | 20 (80.0%)  5 (20.0%) | 40 (75.5%)  13 (24.5%) | 0.663 |
| Multiple types of AHT  No  Yes | 165 (91.7%)  15 (8.3%) | 149 (94.3%)  9 (5.7%) | 0.348 | 43 (91.5%)  4 (8.5%) | 56 (96.6%)  2 (3.4%) | 0.271 | 23 (92.0%)  2 (8.0%) | 48 (90.6%)  5 (9.4%) | 0.839 |
| Calcium channel blockers  No  Yes | 152 (84.4%)  28 (15.6%) | 145 (91.8%)  13 (8.2%) | 0.040 | 38 (80.9%)  9 (19.1%) | 56 (96.6%)  2 (3.4%) | 0.009 | 22 (88.0%)  3 (12.0%) | 46 (86.8%)  7 (13.2%) | 0.884 |
| ACEi or ARBs  No  Yes | 165 (91.7%)  15 (8.3%) | 140 (88.6%)  18 (11.4%) | 0.346 | 43 (91.5%)  4 (8.5%) | 56 (96.6%)  2 (3.4%) | 0.271 | 23 (92.0%)  2 (8.0%) | 43 (81.1%)  10 (18.9%) | 0.220 |
| Beta-blockers  No  Yes | 171 (95.0%)  9 (5.0%) | 155 (98.1%)  3 (1.9%) | 0.125 | 45 (95.7%)  2 (4.3%) | 56 (96.6%)  2 (3.4%) | 0.832 | 24 (96.0%)  1 (4.0%) | 52 (98.1%)  1 (1.9%) | 0.587 |
| Diuretics  No  Yes | 175 (97.2%)  5 (2.8%) | 156 (98.7%)  2 (1.3%) | 0.332 | 46 (97.9%)  1 (2.1%) | 57 (98.3%)  1 (1.7%) | 0.882 | 24 (96.0%)  1 (4.0%) | 53 (100.0%)  0 (0.0%) | 0.147 |

* Correlations between the two groups were examined in chi-squared tests.

HER: human epidermal growth factor receptor. Luminal BC, luminal breast cancer HER2BC, human epidermal growth factor receptor 2-enriched breast cancer. TNBC, triple-negative breast cancer. pCR, pathological complete response. TILs: tumor- infiltrating lymphocytes. AHT: antihypertensive drug. ACEi: angiotensin-converting-enzyme inhibitors, ARBs: angiotensin II receptor blockers.
