# Supplementary material for: Verification of the effects of calcium channel blockers on the immune microenvironment of breast cancer
Source: BMC Cancer. 2019 Jun 24;19:615. doi: 10.1186/s12885-019-5828-5 (PMC6591916; doi:10.1186/s12885-019-5828-5)
Supplement: Supplementary file 4 — Table S3. Difference in clinicopathological features due to calcium channel blockers in hypertension patients. (DOCX 25 kb) [file 12885_2019_5828_MOESM4_ESM.docx]

**Additional file 4: Table S3. Difference in clinicopathological features due to calcium channel blockers in hypertension patients***

| Parameters | All case (*n* = 65) | | | TNBC (*n* =15) | | | HER2BC (*n* =18) | | |
| --- | --- | --- | --- | --- | --- | --- | --- | --- | --- |
|  | Calcium channel blockers | | *p* value | Calcium channel blockers | | *p* value | Calcium channel blockers | | *p* value |
|  | No (*n* =24) | Yes (*n* = 41) |  | No (*n* =4) | Yes (*n* =11) |  | No (*n* =8) | Yes (*n* =10) |  |
| Age (years old)  ≤ 55  > 55 | 5 (20.8%)  19 (79.2%) | 7 (17.1 %)  34 (82.9%) | 0.711 | 3 (75.0%)  1 (25.0%) | 2 (18.2%)  9 (81.8%) | 0.041 | 0 (0.0%)  8 (100.0%) | 2 (20.0%)  8 (80.0%) | 0.201 |
| Tumor size (mm)  ≤ 50  > 50 | 21 (87.5%)  3 (12.5%) | 36 (87.8 %)  5 (12.2%) | 0.972 | 3 (75.0%)  1 (25.0%) | 8 (72.7%)  3 (27.3%) | 0.936 | 8 (100.0%)  0 (0.0%) | 10 (100.0%)  0 (0.0%) | - |
| Skin infiltration  Negative  Positive | 19 (79.2%)  5 (20.8%) | 33 (80.5%)  8 (19.5%) | 0.900 | 4 (100.0%)  0 (0.0%) | 8 (72.7%)  3 (27.3%) | 0.275 | 7 (87.5%)  1 (12.5%) | 9 (90.0%)  1 (10.0%) | 0.876 |
| Lymph node status  Negative  Positive | 8 (33.3%)  16 (66.7%) | 12 (29.3%)  29 (70.7%) | 0.737 | 0 (0.0%)  4 (100.0%) | 4 (36.4%)  7 (63.6%) | 0.183 | 7 (87.5%)  1 (12.5%) | 4 (40.0%)  6 (60.0%) | 0.042 |
| Estrogen receptor  Negative  Positive | 12 (50.0%)  12 (50.0%) | 21 (51.2%)  20 (48.8%) | 0.926 | -  - | -  - | - | -  - | -  - | - |
| Progesterone receptor  Negative  Positive | 17 (70.8%)  7 (29.2%) | 29 (70.7%)  12 (29.3%) | 0.993 | -  - | -  - | - | -  - | -  - | - |
| HER2  Negative  Positive | 15 (62.5%)  9 (37.5%) | 24 (58.5%)  17 (41.5%) | 0.758 | -  - | -  - |  | -  - | -  - | - |
| Ki67  ≤15 %  >15 % | 10 (41.7%)  14 (58.3%) | 18 (43.9%)  23 (56.1%) | 0.863 | 1 (25.0%)  3 (75.0%) | 2 (18.2%)  9 (81.8%) | 0.790 | 5 (62.5%)  3 (37.5%) | 5 (50.0%)  5 (50.0%) | 0.621 |
| Intrinsic subtype Luminal BC  HER2BC, TNBC  Luminal BC | 12 (50.0%)  12 (50.0%) | 21 51.2%)  20 (48.8%) | 0.926 | -  - | -  - |  | -  - | -  - | - |
| Intrinsic subtype HER2BC  Luminal BC, TNBC  HER2BC | 8 (33.3%)  16 (66.7%) | 31 (75.6%)  10 (24.4%) | 0.445 | -  - | -  - |  | -  - | -  - | - |
| Intrinsic subtype TNBC  Luminal BC, HER2BC  TNBC | 20 (83.3%)  4 (16.7%) | 30 (73.2%)  11 (26.8%) | 0.356 | -  - | -  - |  | -  - | -  - | - |
| Objective response rate  Non-Responders  Responders | 3 (12.5%)  21 (87.5%) | 8 (19.5%)  33 (80.5%) | 0.475 | 0 (0.0%)  4 (100.0%) | 4 (36.4%)  7 (63.6%) | 0.183 | 1 (12.5%)  7 (87.5%) | 0 (0.0%)  10 (100.0%) | 0.276 |
| Pathological response  Non-pCR  pCR | 16 (66.7%)  8 (33.3%) | 27 (65.9%)  14 (34.1%) | 0.948 | 2 (50.0%)  2 (50.0%) | 7 (63.6%)  4 (36.4%) | 0.662 | 3 (37.5%)  5 (62.5%) | 2 (20.0%)  8 (80.0%) | 0.440 |
| TILs  Low  High | 13 (54.2%)  11 (45.8%) | 28 (68.3%)  13 (31.7%) | 0.262 | 3 (75.0%)  1 (25.0%) | 9 (81.8%)  2 (18.2%) | 0.790 | 2 (25.0%)  6 (75.0%) | 3 (30.0%)  7 (70.0%) | 0.827 |
| Multiple types of AHT  No  Yes | 20 (83.3%)  4 (16.7%) | 21 (51.2%)  20 (48.8%) | 0.009 | 3 (75.0%)  1 (25.0%) | 6 (54.5%)  5 (45.5%) | 0.510 | 7 (87.5%)  1 (12.5%) | 4 (40.0%)  6 (60.0%) | 0.042 |
| ACEi or ARBs  No  Yes | 8 (33.3%)  16 (66.7%) | 24 (58.5%)  17 (41.5%) | 0.051 | 1 (25.0%)  3 (75.0%) | 8 (72.7%)  3 (27.3%) | 0.109 | 2 (25.0%)  6 (75.0%) | 4 (40.0%)  6 (60.0%) | 0.531 |
| Beta-blockers  No  Yes | 16 (66.7%)  8 (33.3%) | 37 (90.2%)  4 (9.8%) | 0.018 | 3 (75.0%)  1 (25.0%) | 3 (27.3%)  8 (72.7%) | 0.936 | 6 (75.0%)  2 (25.0%) | 10 (100.0%)  0 (0.0%) | 0.105 |
| Diuretics  No  Yes | 20 (83.3%)  4 (16.7%) | 38 (92.7%)  3 (7.3%) | 0.247 | 3 (75.0%)  1 (25.0%) | 10 (90.9%)  1 (9.1%) | 0.459 | 7 (87.5%)  1 (12.5%) | 10 (100.0%)  0 (0.0%) | 0.276 |

* Correlations between the two groups were examined in chi-squared tests.

HER: human epidermal growth factor receptor. Luminal BC, luminal breast cancer. HER2BC, human epidermal growth factor receptor 2-enriched breast cancer. TNBC, triple-negative breast cancer. pCR, pathological complete response. TILs: tumor- infiltrating lymphocytes. AHT: antihypertensive drug. ACEi: angiotensin-converting-enzyme inhibitors, ARBs: angiotensin II receptor blockers.
