# Supplementary material for: Verification of the effects of calcium channel blockers on the immune microenvironment of breast cancer
Source: BMC Cancer. 2019 Jun 24;19:615. doi: 10.1186/s12885-019-5828-5 (PMC6591916; doi:10.1186/s12885-019-5828-5)
Supplement: Supplementary file 5 — Table S4. Univariate and multivariate analysis with respect to DFS. (DOCX 22 kb) [file 12885_2019_5828_MOESM5_ESM.docx]

**Additional file 5: Table S4. Univariate and multivariate analysis with respect to DFS**

|  | Univarite analysis | | |  | Multivariate analysis | | |
| --- | --- | --- | --- | --- | --- | --- | --- |
| Parameters | Hazard ratio | 95% CI | *p* value |  | Hazard ratio | 95% CI | *p* value |
| Age at opetation (yr)  ≤ 55 vs > 55 | 0.728 | 0.436-1.197 | 0.212 |  |  |  |  |
| Tumor size (mm)  ≤ 50 vs > 50 | 3.142 | 1.769-5.348 | <0.001 |  | 2.073 | 1.097-3.784 | 0.256 |
| Skin infiltration  Negative vs Positive | 2.405 | 1.319-4.161 | 0.005 |  | 1.221 | 0.619-2.321 | 0.555 |
| Lymph node status  Negative vs Positive | 2.073 | 1.580-2.731 | <0.001 |  | 2.153 | 1.585-2.942 | <0.001 |
| Estrogen receptor  Negative vs Positive | 0.863 | 0.519-1.416 | 0.561 |  |  |  |  |
| Progesterone receptor  Negative vs Positive | 1.031 | 0.599-1.721 | 0.908 |  |  |  |  |
| HER2  Negative vs Positive | 0.483 | 0.245-0.874 | 0.015 |  | 0.503 | 0.249-0.939 | 0.030 |
| Ki67  ≤15 % vs >15 % | 1.469 | 0.864-2.605 | 0.159 |  |  |  |  |
| Intrinsic subtype Luminal BC  HER2, TNBC vs Luminal BC | 0.864 | 0.522-1.417 | 0.563 |  |  |  |  |
| Intrinsic subtype HER2BC  Luminal BC, TNBC vs HER2BC | 0.684 | 0.328-1.285 | 0.250 |  |  |  |  |
| Intrinsic subtype TNBC  Luminal BC, HER2BC vs TNBC | 1.514 | 0.903-2.493 | 0.114 |  |  |  |  |
| Objective response rate  Non-Responders vs Responders | 0.248 | 0.144-0.447 | <0.001 |  | 0.219 | 0.120-0.414 | <0.001 |
| Pathological response  Non-pCR vs pCR | 0.310 | 0.149-0.583 | <0.001 |  | 0.465 | 0.213-0.926 | 0.029 |
| TILs  Low vs High | 0.508 | 0.295-0.847 | 0.009 |  | 0.858 | 0.483-1.479 | 0.586 |
| Hypertension  No vs Yes | 1.199 | 0.624-2.141 | 0.567 |  |  |  |  |
| Multiple types of AHT  No vs Yes | 1.328 | 0.463-2.999 | 0.559 |  |  |  |  |
| Calcium channel blockers  No vs Yes | 1.150 | 0.506-2.279 | 0.717 |  |  |  |  |
| ACEi or ARBs  No vs Yes | 1.030 | 0.397-2.203 | 0.945 |  |  |  |  |
| Beta-blockers  No vs Yes | 1.381 | 0.337-3.736 | 0.603 |  |  |  |  |
| Diuretics  No vs Yes | 1.252 | 0.071-5.685 | 0.830 |  |  |  |  |

DFS: Disease-free survival. CI: confidence intervals. HER: human epidermal growth factor receptor. Luminal BC, luminal breast cancer. HER2BC, human epidermal growth factor receptor 2-enriched breast cancer. TNBC, triple-negative breast cancer. pCR, pathological complete response. TILs: tumor- infiltrating lymphocytes. AHT: antihypertensive drug. ACEi: angiotensin-converting-enzyme inhibitors, ARBs: angiotensin II receptor blockers.
