# Supplementary material for: Verification of the effects of calcium channel blockers on the immune microenvironment of breast cancer
Source: BMC Cancer. 2019 Jun 24;19:615. doi: 10.1186/s12885-019-5828-5 (PMC6591916; doi:10.1186/s12885-019-5828-5)
Supplement: Supplementary file 6 — Table S5. Univariate and multivariate analysis with respect to DFS in HER2BC. (DOCX 21 kb) [file 12885_2019_5828_MOESM6_ESM.docx]

**Additional file 6: Table S5. Univariate and multivariate analysis with respect to DFS in HER2BC**

|  | Univarite analysis | | |  | Multivariate analysis | | |
| --- | --- | --- | --- | --- | --- | --- | --- |
| Parameters | Hazard ratio | 95% CI | *p* value |  | Hazard ratio | 95% CI | *p* value |
| Age at opetation (yr)  ≤ 55 vs > 55 | 0.337 | 0.086-1.189 | 0.090 |  | 0.583 | 0.138-2.216 | 0.429 |
| Tumor size (mm)  ≤ 50 vs > 50 | 2.700 | 0.580-9.750 | 0.184 |  |  |  |  |
| Skin infiltration  Negative vs Positive | 1.384 | 0.209-5.539 | 0.691 |  |  |  |  |
| Lymph node status  Negative vs Positive | 4.680 | 0.878-86.321 | 0.075 |  | 3.296 | 0.522-64.233 | 0.227 |
| Ki67  ≤15 % vs >15 % | 1.267 | 0.348-5.931 | 0.730 |  |  |  |  |
| Objective response rate  Non-Responders vs Responders | 0.149 | 0.039-0.708 | 0.020 |  | 0.906 | 0.154-5.232 | 0.908 |
| Pathological response  Non-pCR vs pCR | 0.179 | 0.027-0.716 | 0.013 |  | 0.242 | 0.030-1.367 | 0.110 |
| TILs  Low vs High | 0.224 | 0.057-0.792 | 0.021 |  | 0.488 | 0.090-2.840 | 0.406 |
| Hypertension  No vs Yes | - | - | 0.027 |  | - | - | 0.437 |
| Multiple types of AHT  No vs Yes | - | - | 0.262 |  |  |  |  |
| Calcium channel blockers  No vs Yes | - | - | 0.111 |  |  |  |  |
| ACEi or ARBs  No vs Yes | - | - | 0.092 |  | - | - | 1.000 |
| Beta-blockers  No vs Yes | - | - | 0.489 |  |  |  |  |
| Diuretics  No vs Yes | - | - | 0.866 |  |  |  |  |

DFS: Disease-free survival. CI: confidence intervals. HER: human epidermal growth factor receptor. HER2BC, human epidermal growth factor receptor 2-enriched breast cancer. pCR, pathological complete response. TILs: tumor- infiltrating lymphocytes. AHT: antihypertensive drug. ACEi: angiotensin-converting-enzyme inhibitors, ARBs: angiotensin II receptor blockers.
