# Supplementary material for: Verification of the effects of calcium channel blockers on the immune microenvironment of breast cancer
Source: BMC Cancer. 2019 Jun 24;19:615. doi: 10.1186/s12885-019-5828-5 (PMC6591916; doi:10.1186/s12885-019-5828-5)
Supplement: Supplementary file 7 — Table S6. Univariate and multivariate analysis with respect to OS. (DOCX 22 kb) [file 12885_2019_5828_MOESM7_ESM.docx]

**Additional file 7: Table S6. Univariate and multivariate analysis with respect to OS**

|  | Univarite analysis | | |  | Multivariate analysis | | |
| --- | --- | --- | --- | --- | --- | --- | --- |
| Parameters | Hazard ratio | 95% CI | *p* value |  | Hazard ratio | 95% CI | *p* value |
| Age at opetation (yr)  ≤ 55 vs > 55 | 0.746 | 0.377-1.438 | 0.383 |  |  |  |  |
| Tumor size (mm)  ≤ 50 vs > 50 | 2.369 | 1.049-4.862 | 0.039 |  | 1.215 | 0.492-2.790 | 0.660 |
| Skin infiltration  Negative vs Positive | 3.563 | 1.708-7.067 | 0.001 |  | 2.650 | 1.138-5.881 | 0.025 |
| Lymph node status  Negative vs Positive | 1.968 | 1.382-2.818 | <0.001 |  | 2.052 | 1.371-3.112 | 0.001 |
| Estrogen receptor  Negative vs Positive | 0.759 | 0.379-1.469 | 0.417 |  |  |  |  |
| Progesterone receptor  Negative vs Positive | 0.910 | 0.430-1.803 | 0.793 |  |  |  |  |
| HER2  Negative vs Positive | 0.420 | 0.157-0.943 | 0.035 |  | 0.414 | 0.140-1.084 | 0.073 |
| Ki67  ≤15 % vs >15 % | 1.573 | 0.782-3.427 | 0.211 |  |  |  |  |
| Intrinsic subtype Luminal BC  HER2BC, TNBC vs Luminal BC | 0.803 | 0.406-1.549 | 0.515 |  |  |  |  |
| Intrinsic subtype HER2BC  Luminal BC, TNBC vs HER2BC | 0.565 | 0.193-1.333 | 0.207 |  |  |  |  |
| Intrinsic subtype TNBC  Luminal BC, HER2BC vs TNBC | 1.804 | 0.920-3.481 | 0.085 |  | 1.499 | 0.713-3.183 | 0.284 |
| Objective response rate  Non-Responders vs Responders | 0.203 | 0.103-0.421 | <0.001 |  | 0.151 | 0.070-0.334 | <0.001 |
| Pathological response  Non-pCR vs pCR | 0.337 | 0.127-0.757 | 0.007 |  | 0.521 | 0.186-1.267 | 0.156 |
| TILs  Low vs High | 0.677 | 0.338-1.310 | 0.249 |  |  |  |  |
| Hypertension  No vs Yes | 0.875 | 0.328-1.962 | 0.762 |  |  |  |  |
| Multiple types of AHT  No vs Yes | 0.860 | 0.139-2.826 | 0.832 |  |  |  |  |
| Calcium channel blockers  No vs Yes | 0.653 | 0.157-1.825 | 0.454 |  |  |  |  |
| ACEi or ARBs  No vs Yes | 0.572 | 0.093-1.881 | 0.404 |  |  |  |  |
| Beta-blockers  No vs Yes | 1.528 | 0.247-5.040 | 0.585 |  |  |  |  |
| Diuretics  No vs Yes | 2.449 | 0.138-11.364 | 0.440 |  |  |  |  |

OS: Overall survival. CI: confidence intervals. HER: human epidermal growth factor receptor. Luminal BC, luminal breast cancer. HER2BC, human epidermal growth factor receptor 2-enriched breast cancer. TNBC, triple-negative breast cancer. pCR, pathological complete response. TILs: tumor- infiltrating lymphocytes. AHT: antihypertensive drug. ACEi: angiotensin-converting-enzyme inhibitors, ARBs: angiotensin II receptor blockers.
