# Supplementary material for: Verification of the effects of calcium channel blockers on the immune microenvironment of breast cancer
Source: BMC Cancer. 2019 Jun 24;19:615. doi: 10.1186/s12885-019-5828-5 (PMC6591916; doi:10.1186/s12885-019-5828-5)
Supplement: Supplementary file 8 — Table S7. Univariate and multivariate analysis with respect to OS in HER2BC. (DOCX 21 kb) [file 12885_2019_5828_MOESM8_ESM.docx]

**Additional file 8: Table S7. Univariate and multivariate analysis with respect to OS in HER2BC**

|  | Univarite analysis | | |  | Multivariate analysis | | |
| --- | --- | --- | --- | --- | --- | --- | --- |
| Parameters | Hazard ratio | 95% CI | *p* value |  | Hazard ratio | 95% CI | *p* value |
| Age at opetation (yr)  ≤ 55 vs > 55 | 0.389 | 0.051-2.346 | 0.294 |  |  |  |  |
| Tumor size (mm)  ≤ 50 vs > 50 | 1.468 | 0.075-9.960 | 0.742 |  |  |  |  |
| Skin infiltration  Negative vs Positive | 2.028 | 0.101-14.853 | 0.564 |  |  |  |  |
| Lymph node status  Negative vs Positive | - | - | 0.062 |  | - | - | 0.101 |
| Ki67  ≤15 % vs >15 % | 0.378 | 0.049-2.309 | 0.284 |  |  |  |  |
| Objective response rate  Non-Responders vs Responders | 0.384 | 0.056-7.538 | 0.440 |  |  |  |  |
| Pathological response  Non-pCR vs pCR | 0.546 | 0.072-3.299 | 0.503 |  |  |  |  |
| TILs  Low vs High | 0.564 | 0.093-4.302 | 0.542 |  |  |  |  |
| Hypertension  No vs Yes | - | - | 0.100 |  | - | - | 0.166 |
| Multiple types of AHT  No vs Yes | - | - | 0.456 |  |  |  |  |
| Calcium channel blockers  No vs Yes | - | - | 0.245 |  |  |  |  |
| ACEi or ARBs  No vs Yes | - | - | 0.233 |  |  |  |  |
| Beta-blockers  No vs Yes | - | - | 0.572 |  |  |  |  |
| Diuretics  No vs Yes | - | - | - |  |  |  |  |

OS: Overall survival. TNBC, triple-negative breast cancer. CI: confidence intervals. pCR, pathological complete response. TILs: tumor- infiltrating lymphocytes. AHT: antihypertensive drug. ACEi: angiotensin-converting-enzyme inhibitors, ARBs: angiotensin II receptor blockers.
